# Supplementary material for: Investigating molecular markers linked to acute myocardial infarction and cuproptosis: bioinformatics analysis and validation in the AMI mice model
Source: PeerJ. 2024 May 29;12:e17280. doi: 10.7717/peerj.17280 (PMC11143973; doi:10.7717/peerj.17280)
Supplement: Supplemental Information 3 [file peerj-12-17280-s003.docx]

| SupplementTable1 Classification of cluster in AMI. | |
| --- | --- |
| ID | Cluster |
| GSM1167072_Treat | C1 |
| GSM1167078_Treat | C1 |
| GSM1167081_Treat | C1 |
| GSM1167088_Treat | C1 |
| GSM1167073_Treat | C1 |
| GSM1167074_Treat | C1 |
| GSM1167077_Treat | C1 |
| GSM1167079_Treat | C1 |
| GSM1167080_Treat | C1 |
| GSM1167082_Treat | C1 |
| GSM1167083_Treat | C1 |
| GSM1167085_Treat | C1 |
| GSM1167086_Treat | C1 |
| GSM1167087_Treat | C1 |
| GSM1167090_Treat | C1 |
| GSM1167091_Treat | C1 |
| GSM1167093_Treat | C1 |
| GSM1167094_Treat | C1 |
| GSM1167095_Treat | C1 |
| GSM1167096_Treat | C1 |
| GSM1167098_Treat | C1 |
| GSM1167100_Treat | C1 |
| GSM1167101_Treat | C1 |
| GSM1167097_Treat | C2 |
| GSM1167075_Treat | C2 |
| GSM1167076_Treat | C2 |
| GSM1167084_Treat | C2 |
| GSM1167089_Treat | C2 |
| GSM1167092_Treat | C2 |
| GSM1167099_Treat | C2 |
| GSM1167122_Treat | C2 |
