# Supplementary material for: Investigating molecular markers linked to acute myocardial infarction and cuproptosis: bioinformatics analysis and validation in the AMI mice model
Source: PeerJ. 2024 May 29;12:e17280. doi: 10.7717/peerj.17280 (PMC11143973; doi:10.7717/peerj.17280)
Supplement: Supplemental Information 4 [file peerj-12-17280-s004.docx]

| SupplementTable2 Potential therapeutic agents for AMI. | | | |
| --- | --- | --- | --- |
| gene | drug | sources | pmids |
| CBLB | FASUDIL | JAX-CKB | 26177294 |
| CBLB | FLUOROURACIL | JAX-CKB | 24351824 |
