# Supplementary material for: Investigating molecular markers linked to acute myocardial infarction and cuproptosis: bioinformatics analysis and validation in the AMI mice model
Source: PeerJ. 2024 May 29;12:e17280. doi: 10.7717/peerj.17280 (PMC11143973; doi:10.7717/peerj.17280)
Supplement: Supplemental Information 5 [file peerj-12-17280-s005.docx]

| SupplementTable3 The lists of cuproptosis-related genes .. |
| --- |
| Gene |
| NFE2L2 |
| NLRP3 |
| ATP7B |
| ATP7A |
| SLC31A1 |
| FDX1 |
| LIAS |
| LIPT1 |
| LIPT2 |
| DLD |
| DLAT |
| PDHA1 |
| PDHB |
| MTF1 |
| GLS |
| CDKN2A |
| DBT |
| GCSH |
| DLST |
| LIPT11 |
| COX11 |
| UBE2D2 |
| COA6 |
| UBE2D4 |
| PDE3B |
| UBE2D1 |
| H3C1 |
| CD274 |
| MAP2K1 |
| PDK1 |
| SCO1 |
| UBE203 |
| SOD1 |
| SLC25A3 |
| ULK2 |
| CP |
| AOC3 |
| SLC31A2 |
| DBH |
| LOXL2 |
| ULK1 |
| UEFFA |
| MAP2K2 |
| ATOX1 |
| MTCO2P12 |
| TYR |
