# Supplementary material for: Investigating molecular markers linked to acute myocardial infarction and cuproptosis: bioinformatics analysis and validation in the AMI mice model
Source: PeerJ. 2024 May 29;12:e17280. doi: 10.7717/peerj.17280 (PMC11143973; doi:10.7717/peerj.17280)
Supplement: Supplemental Information 6 [file peerj-12-17280-s006.docx]

**Animals and procedures**

For the myocardial infarction (MI) animal models, 6-7-week-old male C57BL/6 mice were purchased from Ningbo University. Experiments were approved by the Committee of Ningbo University School of Medicine (10489) and conducted according to the NIH Guide for the Care and Use of Laboratory Animals. Animals were housed at 23 ± 2℃ with a humidity of 50 ± 5%. Mice were randomly divided into two groups (n >5 for each group): sham group, MI group. See Supplementary Data for the establishment of the MI mouse model.Utilize a rectangular operating table (OT) equipped with a prewarmed heating pad set at a temperature of 37 °C throughout the entirety of the surgical procedure. Prior to commencing the procedure, thoroughly disinfect the operating table using ultraviolet light and a solution consisting of 70% alcohol. To accurately determine the required dosage of anesthetic drugs, carefully weigh each individual mouse. Subsequently, administer anesthesia to the mice via intraperitoneal injection of ketamine and xylazine. To ensure an appropriate level of anesthesia, assess the absence of a withdrawal reflex upon toe pinch stimulation and the absence of blink reflexes. Position the mouse in a supine position on the operating table, taking care to place gauze beneath the head to prevent excessive heat exposure to the eyes. Apply ophthalmic ointment to the eyes to prevent them from drying out.

Shave the fur on the left precordial chest with an electric razor. Use a fur removal cream on the pre-shaved thorax and massage evenly with a sterile cotton swab for ~1 min. Wipe the excess loose fur with gauze. Use povidone-iodine, followed by 70% alcohol to clean the area. Cover the thorax with gauze. Use a 4-0 suture under the upper incisors and secure it to the anchor point (close to the edge of the OT over the nose) to keep the mouth slightly open and facilitate cannulation. Pull the tail to keep the body straight, and secure the tail to the OT using tape. Secure the four limbs and tighten them on the other anchor points. Importantly, do not over-stretch the front limbs; otherwise, respiratory compromise may occur. Use curved forceps and forceps to open the jaw and lift the tongue. Use an illuminator to clearly visualize the throat and glottis. Insert a 22-G cannula gently with a blunted and truncated needle into the trachea through the mouth ~1-cm down the throat. Use one hand to hold the tongue, move it slightly upwards with blunt forceps, and, simultaneously ,use the other hand to gently insert the tube into the trachea. Be careful not to insert the tube into the

esophagus. Remove the needle gently. Check the intubation by placing the tube into the water for bubbles to form before connecting to the ventilator .Connect the endotracheal tube to a ventilator set to 120/min and tidal volume adjusted to 250 µL.

NOTE: The ventilator setting is adjusted by body weight(in general, a higher body weight requires a higher tidal volume).Verify intubation by checking bilateral symmetrical chest expansion. Then, the connection is fixed to the OT with tape to avoid the tube falling off.Place ECG electrodes on the paws and connect them to the ECG recorder. Monitor cardiac electrophysiology throughout the procedure.

Remove the gauze on the thorax. Disinfect again with 70% alcohol for the incision areas using three scrub cycles. Then, cover the mouse with a sterile surgical drape with a hole over the surgical field to reduce contamination of the surgical site. Make an oblique skin incision (0.8-1.0 cm) along the left midclavicular line with a sterile scalpel. Undertake blunt dissection of subcutaneous tissues to expose the ribs underneath. Be careful not to injure vessels, ribs, and lungs. Stop the bleeding by using sterile cotton applicators. Identify and make an incision of about 6-8 mm in the third intercostal space. Then, carry out blunt dissection of tissues in the intercoastal space to open the chest cavity. Be careful not to injure the internal thoracic artery. Use forceps to span the intercostal space. Insert presterilized homemade retractors into the rib cage and pull back to spread the incision to ~6 mm in width. Attach the retractors to the OT with rubber bands. Remove the surrounding tissues carefully to expose the heart fully. Pull off the pericardium gently with curved forceps without injuring the heart. Now a clear view of the heart is available.

Use a dissecting microscope and direct a focused and appropriate light for LAD visualization. Press the site below the chosen ligation position gently to enlarge the LAD temporarily (≤5 s per time). Recheck the LAD in this way. Use a tapered needle (3/8, 2.5 x 5) to pass an 8-0 silk ligature underneath the LAD under a dissecting microscope. Be careful with the needle depth: not too deep to enter the left ventricle and not too shallow to avoid damaging the LAD. Tie the ligature with a loose double-knot. The loop diameter is about 2-3 mm. Place a 2-3 mm PE-10 tubing into a loop parallel to the artery. Tighten the ligature loop gently until it is around the artery and tubing. Then, secure the loop with a slipknot. Take care not to damage the myocardial wall with excessive tightening pressure. NOTE: Ligation is not carried out for the sham-operation group. Confirm cessation of blood flow in the LAD: observe a paler color in the anterior wall of the LV after ligation. In addition, significant ST-elevation within a few heartbeats also indicates occlusion16. If permanent ligation is required (e.g., MI), remove the PE-10 tubing and tie the LAD directly with a knot. Resume the remaining procedure as mentioned in step 4.3 below. Remove the retractors from the incision. Then, close the wound temporarily with a bulldog clamp. Ischemia duration is according to the experimental design. Ensure that the mouse continues to be connected to the ventilator. Close the muscle layers with continuous sutures. Close the skin with a 4-0 nylon suture; continuous sutures and

interrupted sutures are acceptable.

Observe the mouse carefully for signs of recovery from anesthesia, for example, movement of the tail or whiskers. After that, the mouse usually resumes a normal breathing pattern with a respiration rate of around 150 bpm. Extubate the mouse by removing the tube slowly. Monitor the mouse for an additional 3-5 min to ensure respiratory distress is absent. Administer 100 µL of buprenorphine (0.1 mg/mL, s.c.) after the mouse begins to breathe. For the next 24 h, provide an additional dose every 4-6 h. Provide ibuprofen as additional pain relief in drinking water as a 0.2 mg/mL solution for 2 days before and ≤7 days after surgery. Keep the mice warm and reduce mortality risk by using thermal insulation blankets as mice are prone to hypothermia after the anesthesia.

Finally, mice were killed using anesthesia at 3h, 48h and 1 week. There were no mice left in this study and were used for experiments.
